# Patterns of students’ well-being in early adolescence: A latent class and two-wave latent transition analysis

**DOI:** 10.1371/journal.pone.0276794

**Published:** 2022-12-01

**Authors:** Wassilis Kassis, Clarissa Janousch, Petra Sidler, Dilan Aksoy, Céline Favre, Beyhan Ertanir

**Affiliations:** School of Education, University of Applied Sciences and Arts Northwestern Switzerland, Windisch, Switzerland; Lorestan University, ISLAMIC REPUBLIC OF IRAN

## Abstract

Adolescence is a developmental stage with high risks in terms of psychological challenges and adjustments related to subjective well-being. Thus far, the findings reported a general decrease in school-related well-being over time. We considered well-being a multidimensional and latent construct that included both feeling good and functioning well at the individual level, and focused on the interplay between hedonic and eudemonic factors. Data of *N* = 377 high school students in Switzerland were used by conducting an online longitudinal study with two waves. Baseline data was gathered in autumn 2019 and the subsequent time point occurred 1 year later (2020; grades seven and eight). By applying a person-oriented analytical approach via latent class and latent transition analyses, we were able to identify and compare longitudinally three distinct well-being patterns and the respective trajectories. Regarding the distribution of the well-being patterns for both waves, significant changes over time were identified: particularly from wave 1 to wave 2, where there was an increase for the low and high well-being patterns, yet a decrease for the middle pattern. Comparing the stability of the respective patterns over time, the high well-being level showed the highest stability of all identified patterns. Multinomial logistic regression of covariates to the identified latent status membership established for both waves showed low but significant effects of socio-demographic variables. At wave 1, having a migration background was associated with a significant increase of being in a low versus high well-being level pattern. At wave 2, being female was associated with a significant increase of being in a low versus high and in a middle versus high well-being pattern.

## Introduction

Since the beginning of the 2008 recession, the decline in all age groups’ well-being level [1, 2] has been the most abrupt in Europe, including Switzerland [3]. However, before the onset of the economic crisis, scholars had tentatively started focusing on subjective and psychological approaches, particularly on the role of schools in adolescent students’ well-being [4–6].

Despite the bulk of literature on adolescent students’ well-being, the respective changes during adolescence [7], and the general academic interest in adolescent students’ well-being in recent decades [8], a vivid debate is continuing about the definition and measurements of well-being [9–11]. Particularly, the discussion on the relations between various well-being components is still ongoing [12], while acknowledging that adolescents experience lower levels of well-being than younger children or adults [1, 13]. As adolescent students’ well-being is one of the most crucial predictors of adolescents’ mental health [1, 4], and additionally because of adolescence itself as a psychologically risky developmental stage, adolescent students’ well-being is considered the psychological cornerstone of a healthy transition from childhood to early adulthood [3, 8]. Especially during the outbreak of the COVID-19 pandemic, it has been demonstrated that mental health status and the associated adolescent student’s well-being requires close attention (74, 75).

### Well-being components of adolescents at school

As there is no common definition, we consider adolescent students’ well-being a multidimensional and latent construct that includes aspects of adolescents feeling good (hedonic concept), the presence of positive and the absence of negative affect, and functioning well (eudemonic concept) at school [14, 15]. The variety of theoretical concepts and empirical measures on well-being impedes comparing studies [16]. Most definitions are based on describing the concept rather than its components [10], which makes it difficult to draw conclusions, run comparisons, and create broad interventions [17]. Even though policymakers and/or academics commonly use the term, it is still inconsistently defined [11]. Therefore, in what follows, we focus on hedonic and eudemonic theories in defining students’ well-being at school.

#### Specifications on adolescent students’ well-being

The two concepts, i.e., hedonic and eudemonic theories, are the backbone of understanding adolescent students’ well-being [18] and have been empirically confirmed as distinct but still closely related theories [19] with long- and short-term benefits [20, 21]. Therefore, it is highly important for adolescent students’ well-being to be conceptualized not only on the side of adolescents feeling good (hedonic concept), but also to perform and function well in academic contexts (eudemonic concept). Similar to adults, adolescent students’ well-being refers to their life experiences because it is related to their social, emotional, cognitive, and academic performance–being far more than just a general satisfaction with school grades [22]. Thus, the interrelations of external and internal factors regarding adolescent students’ well-being play an essential role that goes far beyond a general well-being at school [23–25]. Therefore, hedonic and eudemonic well-being is expected to be correlated yet distinct aspects.

#### The hedonic aspect of well-being

The hedonic theories of well-being for adolescent students emphasize striving for positive experiences and consist of affective and cognitive components, such as higher levels of self-esteem and life satisfaction [23]. The association between self-esteem and well-being in adolescence is well documented [26]. High school students with a higher sense of self-esteem feel less threatened by school challenges [27] and develop through this process a sense of agency regarding their school behavior. The state of self-esteem being reported as an emotional hedonic component is among the most decisive determinants of well-being in adolescence [28–30]. The well-being of adolescent students is not only an extract of pleasant emotions at school but also relates to general life satisfaction [31], which is an essential cognitive hedonic component of well-being [32]. Life satisfaction relates to a broad conceptualization concerning adolescent students’ assessment of their happiness with their lives as a whole [33, 34].

#### The eudemonic aspect of well-being

Well-being at school goes beyond the experience of an individual’s satisfaction. It also includes a eudemonic side, which embodies positive social skills promoting a person’s positive functioning in their respective environment at school [14, 18, 35–37]. Dimensions are mastery at school, such as having good grades, high self-efficacy, and fulfilling basic psychological needs. The latter has its roots in *Self-determination Theory*, which includes positive relations with others, autonomy, and growth in academic competence as being essential to well-being at school [23, 38, 39]. Prominently, Deci and Ryan [38] have identified autonomy, experiences of being competent, and social relatedness as basic psychological needs. Deci and Ryan’s self-determination theory of motivation focuses on these three primary psychological needs; the satisfaction of these needs represents a decisive requirement for intrinsic motivation. Coincidently, meeting basic psychological needs results in developing autonomy, competence, and social relatedness. The psychological need of competence is focused on reliable instrumentalities leading to specific outcomes [38, 40]. The need for autonomy focuses on adolescent students’ aspirations to experience the self as the origin of their actions at school [23]. Adolescent students’ need for social relatedness encompasses the universal urge to be connected, to interact with, and to experience interrelated caring during their studies. In general, adolescent students need to feel securely connected with and to feel understood by other people [40]. Because of these interrelations between internal and external school factors within the frame of the eudemonic concept, theoretical and empirical constructs such as self-determination [40], are considered crucial pillars of adolescent students’ well-being [14].

Bandura [41] has defined perceived self-efficacy within the *Social Cognitive Theory* as a generalized concept of behavioral expectations based on mastery experience. According to Bandura [41], perceived self-efficacy is based on four sources of information: mastery experiences, vicarious experiences, verbal persuasion, and physical or affective states, whereby mastery experiences are assumed the most important. Bandura’s [42] theory underlines that perceived self-efficacy influences both motivation and volition phases during the acting process. Consequently, adolescent students tend to set higher goals and be more efficient and realistic in planning their actions. Therefore, perceived self-efficacy enhances well-being [43]. Whereas Bandura [41, 42] described perceived self-efficacy as a domain-specific belief, Jerusalem and Schwarzer [44, 45] developed a scale for measuring general perceived self-efficacy as the universal belief for being able to cope with life challenges. This scale, as outlined in the current study, is more adequate if global concepts are predicted [46]. A higher level of perceived self-efficacy is considered favorable for setting and achieving goals and is relevant for autonomy and competence within the self-determination theory.

#### Stability of adolescent students’ well-being

Although identifying adolescent students’ well-being patterns at a given time is an important first step, knowing whether, why, and how these patterns change longitudinally is essential to design school-specific prevention and intervention programs. Research indicates that there is a decreasing-with-age tendency of well-being during adolescence [47, 48]. More recent studies verified this decreasing trend: For example, Casas and González [48] found evidence for decreases in self-reported well-being during adolescence in a sample of *N* = 48,040 participants–aged between 7 and 14 –across 15 countries. They additionally argued that the respective results and the fluctuating scores differ significantly from country to country. Moreover, the authors presented evidence for a much earlier onset of the decreasing trend, showing that in most countries a well-being decrease starts at around 10 years of age. Findings of decreasing well-being have been also observed in another two-year follow-up study of adolescents, which was specifically focused on younger adolescents from ages 12 to 14 [49]. However, the findings of decrease were only reported for school-related well-being, whereas the well-being concerning health and friends remained stable. In addition, González-Carrasco et al. [7] also observed a significant decrease in well-being during adolescence using data from a sample of *N* = 940 Spanish students aged 9–16 (mainly 10–14). In contrast, there are also studies reporting mixed results with no changes within adolescence [e.g., 50, 51].

Consequently, it is apparent that well-being development and the well-being rates throughout adolescence remain unclear across countries, especially also due to the fact that very different scales are used. Casas and González [48] identified 13 different psychometric scales. In addition to the discussion concerning the conceptualization of well-being, there is still an unresolved question in terms of the well-being’s stability throughout adolescence. Even though, there is evidence for both—instability versus stability—there is more evidence for instability, indicating a significant change across the childhood and adolescence period. To address this issue, this study aimed to examine the combined contribution of eudemonic and hedonic well-being factors in predicting subjective adolescent students’ well-being patterns over time by applying Latent Transition Analysis (LTA). LTA is a longitudinal analysis technique which characterizes transitions over time [52]. Using this person-oriented procedure, we expect to estimate and understand the respective adolescent students’ continuity of well-being levels at two time points, whether the transition is developmentally forward (e.g., transition to higher well-being levels) or backward (e.g., transition to lower well-being levels). This methodology allows grouping of subjects into distinct classes according to the adolescent students’ well-being indicators included in the analysis, and then estimates the probability that a particular subject (thus also a person-oriented method) is a member of that class [52, 53].

Moreover, we need to understand the interplay between covariates and well-being level predictors to the respective well-being patterns and the potential changes. Research suggests that low socio-economic status [5] and migration background [54–57] predict adolescent students’ well-being. The underlying assumption to this prediction is connected to the fact of a lower extent of available social and personal resources and to higher societal discrimination [5, 56, 57]. Additionally, female adolescent students show higher levels of internalizing symptoms than male students [57–59], which is associated with lower levels of well-being. Therefore, it is hypothesized that these socio-demographic factors might influence the membership in different groups that show each pattern of well-being and its stability and change one year later.

It is commonly hypothesized [47–49], but still not convincingly shown [50, 51], that well-being in early adolescence will drop over time. Thus, this study was conducted to fill these gaps in knowledge by discovering well-being patterns over time. Also, research suggests that female gender [55, 57] and a migration background [55–57] might mitigate membership in higher well-being patterns. In addition, school students enrolled in lower school level classes who are from families with a lower socio-economic background [5, 54] are expected to have a higher probability of membership in lower well-being patterns compared to students enrolled in higher school level classes who are from families with a higher socio-economic background.

## Materials and methods

### Participants

The representative convenient random sample data of *N* = 377 high school students out of 33 school classes in German-speaking Northwestern Switzerland was collected anonymously by completing an online questionnaire twice in an interval of one year. Parents and teachers also received questionnaires parallel to the adolescents. These questionnaires are not discussed in this paper, only sociodemographic information from the parents’ questionnaires was used. Wave 1 was in autumn 2019, with seventh-grade adolescent students, and wave 2 in autumn 2020 with the same participants one year later. Consent forms were obtained from students and their parents. No incentives were given. An ethics research committee at the University of Zurich in Switzerland authorized the project. On the day of the study, the research team members gave students a short oral introduction to the survey and the students completed the questionnaire in about 35‒60 minutes. The overall sample average age at wave 1 was *M_age_wave* 1 = 12.67 (*SD*_age_wave 1 = .68) and at wave 2, *M_age_wave* 2 = 13.60 *(SD_age_wave* 2 = .67). At wave 1, 47% of participants (*n* = 167) were female. At wave 2, 45% of participants (*n* = 143) were female and 0.1% (*n* = 2) identified as neither female nor male. At wave 2, 47% (*n* = 176) participating students had a migration background.

In terms of attrition from wave 1 to wave 2, there were no significant differences in terms of the tested socio-demographic variables between wave 1 (*n* = 377) and wave 2 (*n* = 319) participants (gender_*t*(355) = .904, *p* >.05.; migration background_ *t*(375) = -1.536, *p* >.05.; school-level_*t*(375) = 1.588, *p* >.05). Due to this, the two samples are comparable even though some participants were lost due to moving away or dropouts.

### Measures

#### The six latent class/latent transition indicators

*The three hedonic indicators*. The satisfaction with life scale: This scale [31] measures the subjective criteria for satisfaction with life on five items using a seven-point Likert scale (range: 1 = *totally disagree* to 7 = *completely agree*) and displayed a high reliability for both waves (Cronbach’s Alpha (α) _wave 2 = .82; αwave 1 = .80). Example: “For most things, my life is close to my ideal.”

Self-esteem: The Rosenberg Self-Esteem Scale [60] on assessing an individual’s global worthiness evaluation is a 10-item scale with higher scores indicating higher self-esteem. The items were rated on a four-point Likert scale ranging from 1 = *not at all* to 4 = *completely true* (α_wave 2 = .85; α_wave 1 = .82; e.g., “I wish I could respect myself more.”).

Well-being: The five-item scale is based on Makarova’s [61] well-being scale (α_wave 2 = .71; α_wave 1 = .68; e.g., “How do you feel at home?”) and was measured on a four-point Likert scale (range: 1 = *not well* to 5 = *well*).

*The three eudemonic indicators*. Self-efficacy: The General Self-Efficacy Scale is a psychometric scale Schwarzer and Jerusalem [45] developed and is designed to assess optimistic self-belief regarding coping with various challenging demands in life (e.g., “I am confident that I could deal efficiently with unexpected events”). The 10-item scale (α_wave 2 = .89; α_wave 1 = .88) was measured on a four-point Likert scale (range: 1 = *not true* to 4 = *completely true*).

Self-determination: Following Deci and Ryan’s [38, 39] Self-Determination Theory (SDT) on human basic psychological needs, we measured the three subscales *autonomy*, *competence*, and *relatedness* on short scales with three items each (e.g., “I was free to do things in my own way”). The nine-item scale (α_wave 2 = .79; α_wave 1 = .78) was measured on a four-point Likert scale (range: 1 = *not true at all* to 4 = *completely true*).

Satisfaction with grades at school: Satisfaction with grades at school is a single-item indicator addressing the individual student’s assessment of their grades and was measured on a four-point Likert scale (range: 1 = *not at all satisfied* to 4 = *completely satisfied*).

#### Predictors

*Gender*. Students’ gender was assessed with three response options (0 = *male*, 1 = *female*).

*Socio-economic status (SES)*. Students’ SES was used as a proxy for students`socioeconomic background. Information on parental education mainly was gathered from the two questions of the parental questionnaire: “What is the highest level of school education that you have completed?” (ranging from 1 = *General High-school Certificate*/*Vocational Highschool Certificate/Primary Education Diploma* to 5 = *I did not finish Primary School*) and additionally “Have you completed University, ETH, Higher Education School of Technology, Higher Education Pedagogical School (graduate with license to practice a profession, diploma, masters, bachelors, teaching diploma)” (response option = *yes/no*). Using this information, the final SES variable was created, ranging from 0 = *University Degree/Higher Education* to 5 = *Has not finished primary school*. Primarily, the information of T0 data was used, and in cases of missing values, wave 1 data was also considered. In addition, if there still was missing information concerning the parental education, more information was gathered from the student questionnaire’s item “What is the highest completed qualification or school education of your parents?” (which was assessed for each parent separately, in cases of differing qualifications the highest one was selected).

*Migration background*. Not having a migration background means the student and both of their parents were born in Switzerland and all three possess only the Swiss nationality. Having a migration background is operationalized such that one or more of the aforementioned conditions do not apply (0 = no migration background, 1 = with migration background).

*School level*. In Switzerland, adolescents in high schools are divided based on performance and teacher recommendations on mainly three school levels (lower, higher secondary school level, and Gymnasium). Our study was on the lower and higher secondary school level (1 = lower school level, 2 = higher school level).

### Analytic strategy

This study’s aim was two-fold. First, to test the introduced well-being conceptualization by using both eudemonic and hedonic aspects of well-being. Second, assessing adolescent students’ well-being patterns over time [62].

LCA and LTA are typological person-oriented approaches applied to empirically classify latent variables to subgroups based on observations that appear to be similar [53, 63, 64]. The individuals were assigned to the different patterns based on their posterior probabilities for class membership.

Therefore, this study’s statistical analysis was conducted in four steps: First, wave 1 versus wave 2-survey differences in the six applied measures (life satisfaction, self-efficacy, self-esteem, self-determination, well-being, and satisfaction with grades at school) were examined using *t*-tests. Second, adolescent students’ well-being classes were identified separately by computing latent class analyses using six classification variables each for wave 1 and for wave 2. Additionally, invariance analysis across time was applied to ensure for both study waves the reliability for the identified number of well-being patterns (configural invariance) as well as the same relevance of the well-being patterns (metric invariance). Third, we ran LTA to indicate significant differences in the longitudinal classification variables on the identified well-being patterns. Forth, gender, migration-background, socio-economic level, and school level variables were included as predictors to multinomial logistic regression analyses to predict the identified latent status membership. For all conducted LCA/LTA we used *M*plus version 8.6 [65]. For the multinomial regression, SPSS 25 was used.

## Results

### Analytic step one: Differences of all measures between the two waves

We ran *t*-tests (see Table 1) to analyze mean differences between the two waves of the eight applied measures for our sample (Wave 1_*n* = 377, Wave 2_*n* = 319). Overall, only small, a minor effect on decreasing well-being, or no effects on the measures were displayed.

**Table 1:** Wave 1 and wave 2 sample mean levels (and standard deviations) of all observed variables.

Variables	Range	Wave 1	Wave 2	Cohen’s *d*
		*M* (*SD*)	*M* (*SD*)	
Life Satisfaction	1–7	5.00 (1.23)	4.89 (1.32)	-
Self-Efficacy	1–4	2.85 (.52)	2.84 (.54)	-
Self-Esteem	1–4	2.95 (.53)	2.93 (.60)	-
Self-Determination	1–4	2.89 (.44)	2.87 (.45)	-
Well-being	1–5	4.48 (.52)**	4.39 (.58)	-.16
Satisfaction with grades at school	1–4	2.77 (.74)	2.68 (.78)	-

Notes. Wave 1_*N* = 377, Wave 2_*n* = 319;

* = *p* < .05.,

** = *p* < .01., between Wave 1 and Wave 2.

By testing the inter-correlations of all variables (see Table 2) it has been assured that no multi-collinearity problems existed in our analysis. We found low to moderate inter-correlations except for two measures in wave 2 (Self-Determination to Self-Esteem, *r* = .63).

**Table 2:** Correlation between measures.

	1.	2.	3.	4.	5.	6.	7.	8.	9.	10.	11.	12.
1. Life Satisfaction_wave 1	1											
2. Self-Efficacy_wave 1	.34***	1										
3. Self-Esteem_wave 1	.35***	.41***	1									
4. Self-Determination_wave 1	.27***	.33***	.49***	1								
5. Well-being_wave 1	.37***	.28***	.40***	.40***	1							
6. Satisfaction with grades at school_wave 1	.25***	.25***	.31***	.32***	.15**	1						
7. Life Satisfaction_wave 2							1					
8. Self-Efficacy_wave 2							.36***	1				
9. Self-Esteem_wave 2							.54***	.43***	1			
10. Self-Determination_wave 2							.51***	.39***	.63***	1		
11. Well-being_wave 2							.52***	.32***	.38***	.36***	1	.
12. Satisfaction with grades at school_wave 2							.39***	.34***	.39***	.40***	.34***	1

Note:

*** *p* < .001,

** *p* < .01.

The attrition rate from wave 1 (*n* = 377) to wave 2 (*n* = 319) of only 18.2% is low, considering the ongoing Covid-pandemic. No significant differences were found regarding the tested socio-demographic variables between wave 1 and wave 2 gender_*t*(298) = -1.672, *p* > 0.05; and migration background *t*(377) = −1.373, *p* > 0.05. Due to this, the two samples are comparable.

### Analytic step two: Identifying well-being patterns separately for wave 1 and for wave 2 by LCA

In step two, we identified adolescent students’ well-being patterns by computing two separate LCAs (LCA for wave 1 and for wave 2) using six classification variables. A separate set of LCAs for each wave were performed to determine the optimal number of classes at each time point. The 377 individual cases from wave 1 and the 319 cases from wave 2 were assigned separately to a group based on their response similarity in the measured six indicators. Thus, individuals in each group shared the same pattern of well-being [52]. The LCA was conducted for a range of two to five latent classes. The main aim was to determine significantly distinct well-being classes.

The estimated models were non-nested models; therefore, the procedures chosen for model selection were the sample-adjusted Bayesian information criterion (aBIC) indicating goodness of fit. A lower value indicated a more appropriate fit, and entropy [53] indicated the certainty in the estimation, with values above 0.7 considered sufficient [63, 64]. However, the final model for an LCA (i.e., how many classes) was chosen based on a mix of statistical indicators and extant theoretical considerations [53]. We applied different criteria for the model selection. First, the entropy value indicated the certainty in the estimation with values above 0.7 considered sufficient [64]. Second, with information criteria, such as Akaike information criterion (AIC), Bayesian information criterion (BIC), and Sample-Adjusted BIC (ABIC), with the smaller values fitting the model better [64]. For the LCA, we additionally applied model fit criteria as the Vuong-Lo-Mendell-Rubin Likelihood Ration test (LMR-LRT), the Lo-Mendell-Rubin Adjusted Likelihood Ratio test (aLMR-LRT), and the Bootstrapped Likelihood Ratio test (BLRT) with significant *p*-values indicating an improvement to the previous model with k-1 classes [64].

**Fig 1:**
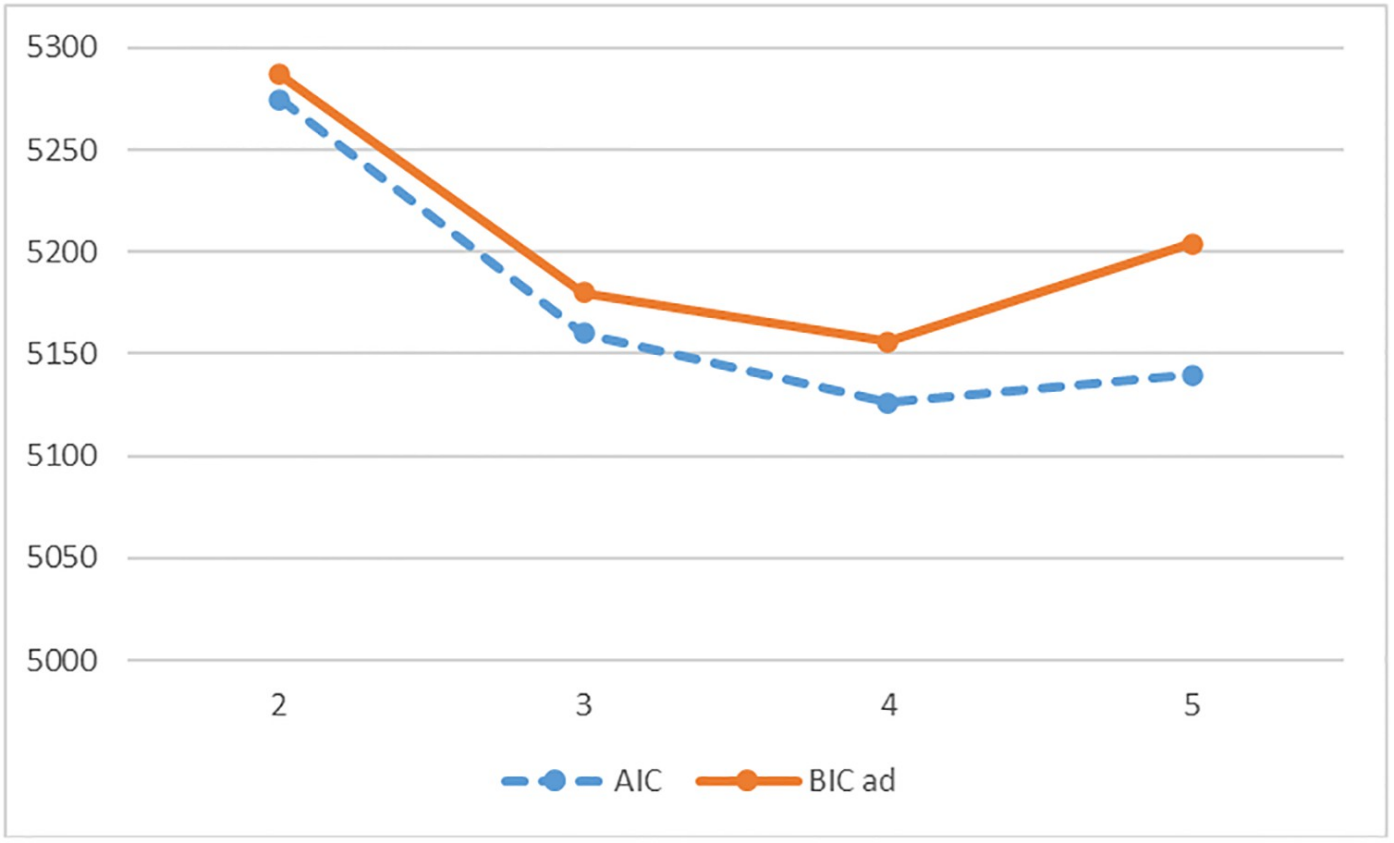
LCA elbow plot on AIC and aBIC of wave 1 well-being at school.

Based on the six indicators (life satisfaction, self-efficacy, self-esteem, self-determination, well-being, and satisfaction with grades at school), a series of LCAs was applied to group students into empirically distinct well-being classes. For wave 1, the aBIC scores dropped between the two and three class solutions and the still-significant tests (VLMR, aLMR, and the bootstrap likelihood ratio test (BLRT)) indicated an improvement supporting a three over two classes solution. Between classes three and four there was a aBIC rise (ΔBIC = 3), and all three performed tests (VLMR, aLMR, and BLRT) indicated no improvement between class three to class four solution. Therefore, a three classes solution was selected for wave 1 (see Fig 1).

For wave 2, the differences between the aBIC scores for the two, three, four, and five class solutions were small, which suggested weak evidence [63] for a higher class-number solution. The highest drop supporting the elbow effect between the classes was noticed between the class two to class three solution (ΔaBIC = 23).

The three performed tests (VLMR, aLMR, and BLRT) were not indicative for a specific solution when related to the respective adjusted BIC drops. Given the above-reported criteria and the rule of deference to more constrained and parsimonious models [64], the three-class solution also was selected for wave 2 (see Table 3). Model fit statistics to select the number of classes of well-being at school are also displayed in Table 3.

**Table 3:** Latent class analysis model fit statistics to select the number of classes of well-being at school for both waves sequentially.

Wave 1								Wave 2						
Classes	AIC (dF)	aBIC	VLMR	aLMR	BLRT	Entropy	Samples	AIC (dF)	aBIC	VLMR	aLMR	BLRT	Entropy	Samples
**2**	2729 (13)	2738	< .001	< .001	< .001	.75	210/ 167	2578 (13)	2588	< .001	< .001	< .001	.83	201/ 176
**3**	2707 (20)	2723	< .001	< .001	< .001	.79	90/ 113/ 174	2555 (20)	2570	>.05	>.05	< .001	.71	145/ 117/ 115
**4**	2710 (27)	2730	>.05	>.05	>.05	.77	118/ 95/ 71/ 93	2542 (27)	2563	< .001	< .001	< .001	.76	137/ 34/ 127/ 79
**5**	2712 (34)	2738	< .001	>.05	>.05	.80	55/ 115/ 80/ 29/ 98	2547 (34)	2573	>.05	>.05	>.05	.80	48/ 127/ 20/ 107/ 75

Note. AIC = Akaike information criterion; aBIC = sample-size adjusted Bayesian information criterion; VLMR = Vuong–Lo–Mendell–Rubin Likelihood Ratio Test; aLMR = Lo–Mendell–Rubin Adjusted LRT Test

**Fig 2:**
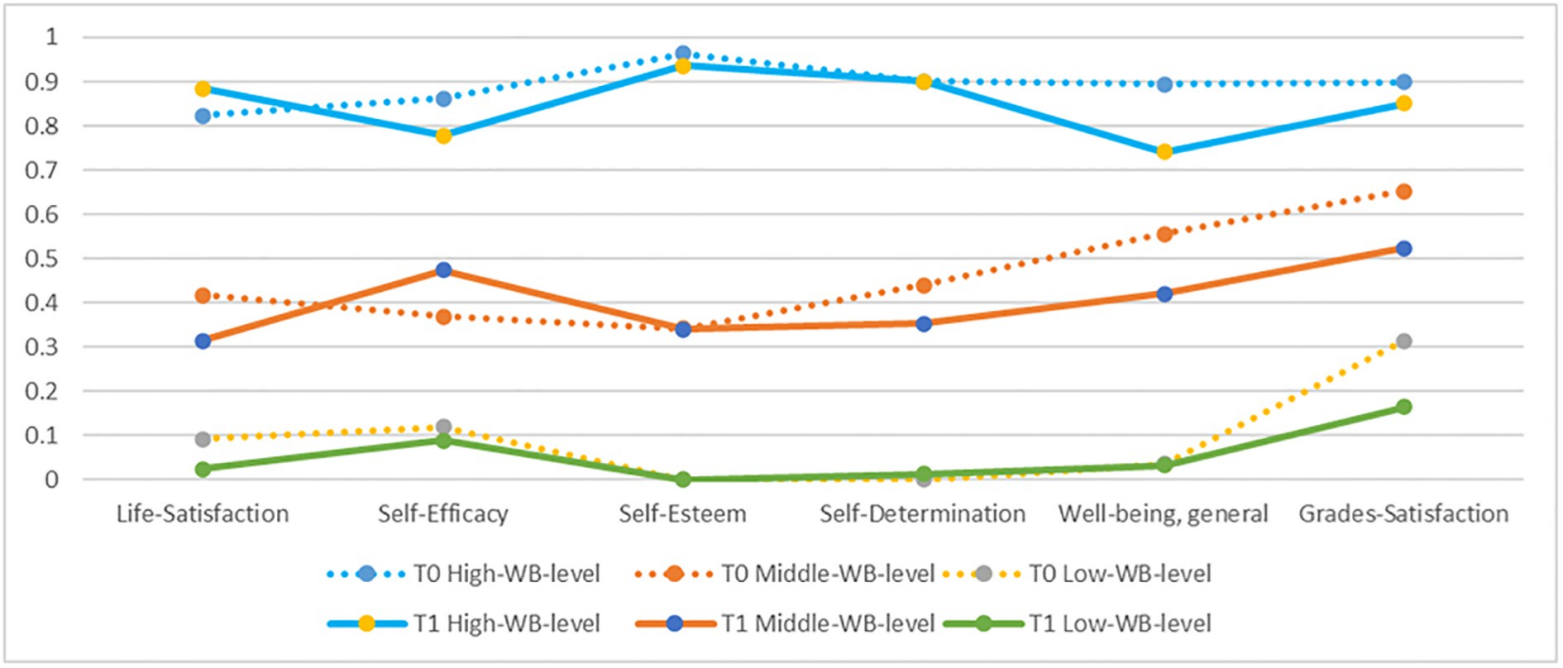
Pattern plot for both waves with class-specific probabilities of the respective high level for the indicators.

Regarding the distribution of the six indicators on the three identified classes for both waves (see Fig 2), we identified vast similarities: A low (wave 1 = 24.1%; wave 2 = 35.5%), a middle (wave 1 = 46.91%; wave 2 = 31.0%), and a high level (wave 1 = 28.9%; wave 2 = 33.4%) well-being class at both time points was detected. The indicators’ probabilities (see Fig 2) on the respective levels are highly comparable on all six indicators, supporting the chosen classes solution for both waves. For both waves the students’ immense well-being differences when comparing the respective low- to the high-level patterns has been identified. For example, see the category 2 (high level of the respective indicator) probabilities comparison of the six indicators (see Fig 2), especially the results on the self-esteem of low well-being patterns for wave 1 and wave 2 (both 0%) and high well-being patterns for wave 1 (96.4%) and wave 2 (93.6%). Likewise, the results are indicative on self-determination: low well-being pattern for wave 1 (0%) and wave 2 (1.4%) and high well-being patterns for wave 1 and wave 2 (both 90%).

Based on the identified three well-being patterns for both waves, we tested for measurement invariance [64] across time in the number of well-being patterns (configural invariance) that could be analyzed for wave 1 and wave 2. Additionally, it was tested whether the loadings on the respective latent classes were invariant, thus ensuring that the factors’ structures, that is, the three well-being patterns, were the same for both study waves (metric invariance). When testing for metric measurement invariance, we identified a nonsignificant chi-square difference test (Δchi^2^ [18] = 27.62, *p* > 0.05.), thereby establishing the same relevance for the three well-being patterns for both study waves. Ensuring metric invariance was the first approach necessary for comparing the three well-being patterns over time.

To summarize the invariance testing results, we found the same number of well-being patterns and well-being dimensions present across both waves over a one-year period. This indicates that the three introduced and empirically analyzed well-being patterns provided an empirically reliable measure over time.

Having established this structure similarity for both waves, the third analysis step on testing stability and change among different patterns of well-being by applying LTA can be approached.

### Analytic step three: LTA to indicate significant differences in the longitudinal classification variables

In step three, an LTA was applied to indicate significant differences in the longitudinal classification variables on the identified well-being patterns. LTA, the longitudinal extension of LCA, is a statistical tool that can fulfill the needs of modeling adolescent well-being transitions over time [64]. It can estimate the continuity of well-being at adjacent time points, whether the transition is forward (e.g., transition from a lower well-being pattern to a higher) or backward (e.g., from a higher well-being pattern to a lower). After determining the optimal number of classes separately at each time point to be three (see analysis step two), we performed an LTA to estimate the probabilities of well-being pattern changes over time from one latent class to another [64]. In this statistical step, change is represented by the probability of transitioning to a latent well-being status at wave 2, given latent status membership at wave 1 [64]. Also, it explores whether the same latent status can be identified in both wave 1 and wave 2 [64].

We ran an LTA by using the previously mentioned six classification variables (for model fits see Table 4). The LTA was conducted for a range of two to five latent classes to test if the conditional response probabilities had been constrained to be time-invariant.

**Table 4:** Latent transition analysis model fit statistics to select longitudinally the number of classes of well-being at school.

Classes	AIC (df)	aBIC	Entropy	Samples
**2**	5275 (15)	5287	.80	c1: 196/ 181; c2: 210/ 167
**3**	5160 (26)	5180	.76	c1: 102/ 25/ 50; c2: 1/ 87/ 21
**4**	5126 (39)	5156	.78	c1: 87/ 25/ 1/ 10; c2: 1/ 65/ 1/ 22
**5**	5140 (84)	5204	.85	c1: 82/ 26/ 12/ 4/ 7; c2: 0/ 67/ 19/ 5/ 54

Note. AIC = Akaike information criterion; aBIC = adjusted Bayesian information criterion

The elbow profile plot on the sample-adjusted aBIC and on the Akaike information criterion (AIC) when testing for two to five profiles (see Fig 2) indicates by both information criteria a three classes solution. The detected samples for the respective solutions (see Table 5) support this with the four class and five class solutions having numerous sub-samples with far too few (< *n* = 20) allocated students to the respective sub-samples (four classes solution with 50% and five classes solution with 80% of the sub-samples having less than *n* = 20 students). Due to this and the rule of deference to more constrained and parsimonious models, a three-class solution was selected for the longitudinal comparison via LTA.

**Table 5:** Estimated longitudinal probabilities of the three well-being levels by latent transition analysis.

Level Well-Being	Wave 1	Wave 2	Δ W2-W1
Low Well-Being	23.9%	31.0%	+ 7.1%
Middle Well-Being	46.1%	30.5%	- 15.6%
High Well-Being	30.0%	38.5%	+ 8.5%

Regarding the distribution of the three classes for both waves (see Table 5) we identified significant changes over time, particularly an increase in the low well-being pattern (of 7.1% from wave 1 to wave 2), a decrease in the middle pattern (of 15.6% from wave 1 to wave 2), and an increase in the high well-being pattern (of 8.5% from wave 1 to wave 2).

**Fig 3:**
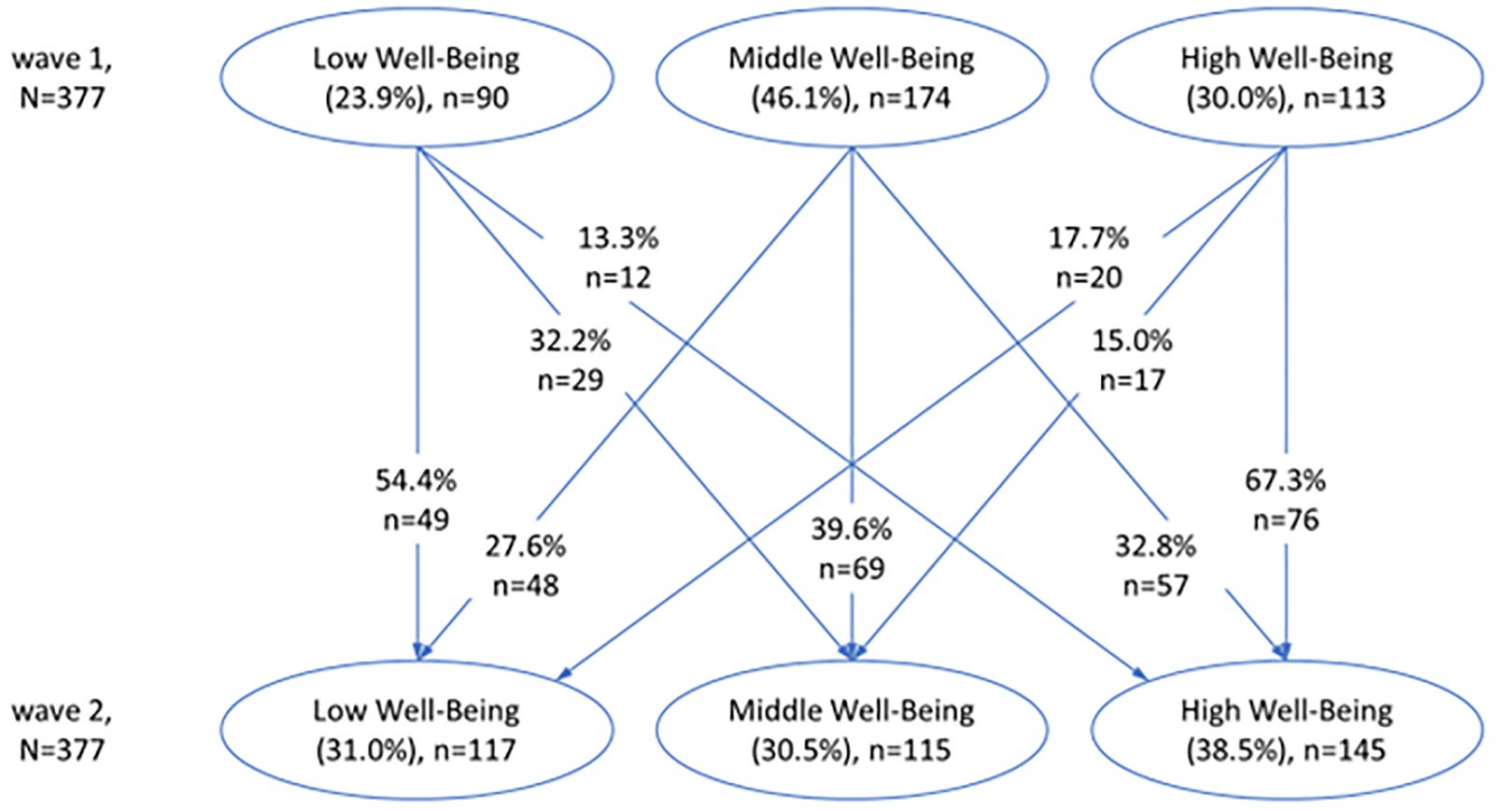
Transition over time among different patterns of well-being levels N = 377 participants followed-up, in parentheses, estimates of status membership probabilities.

In terms of comparing the respective groups’ stability over time, a heterogeneous picture can be identified (see Fig 3) as just 39.6% (*n* = 69) of the students who were in the middle well-being class at wave 1 were still in the middle well-being class at wave 2. In comparison, 54.4% (*n* = 49) of the students who were in the low well-being class at wave 1 were still in the low well-being class at wave 2. Further, 67.3% (*n* = 76) of the students who were in the high well-being class at wave 1 were still in the high well-being class at wave 2, showing the highest stability of all identified patterns.

The students at wave 2 of the low well-being level at wave 1 mostly transitioned to the middle (32.2%) (*n* = 29) and just 13.3% (*n* = 12) to the higher well-being level (see Fig 3). The probability of a downward transitioning from the middle well-being at wave 1 to a low pattern at wave 2 was 27.6% (*n* = 48) and the forward transition to the high well-being level at wave 2 was 32.8% (*n* = 57). Interestingly, within the studied one-year time period and the comparison made from grade seven to grade eight, the backward transition from the high-level pattern at wave 1 to a middle level at wave 2 was 15.0% (*n* = 17) and 17.7% (*n* = 20) to the low-level well-being.

### Analytic step four: Multinomial logistic regression with factors predicting the latent status membership

After identifying the classes for both waves, a multinomial logistic regression was applied. Our analysis included socio-demographical covariates for both waves that could plausibly relate to well-being pattern variations at school (see Table 6): Gender, migration background, socio-economic status, and school level were included as socio-demographic predictors to the identified latent status membership.

**Table 6:** Wave 1& 2, multinomial logistic regression of socio-demographic covariates, resilience, and depression/anxiety to the identified latent status membership: Parameter estimates of both models.

Socio-demographic factors					Socio-demographic factors		
Wave 1					Wave 2		
Well-being level^1^		B (SE)	Wald statistic	OR	B (SE)	Wald statistic	OR
Reference Pattern «High» vs. Pattern «Low»	Intercept	-.57 (.94)	.37	-	-2.42* (.96)	6.35	-
	Gender (1 male; 2 female)	.06 (.33)	.03	1.06	.84* (.33)	6.47	2.31
	Migration background (0 no MB, 1 with MB)	.99** (.37)	7.17	2.71	.52 (.34)	2.34	1.69
	School Level (1 lower; 2 higher)	.08 (.37)	.04	1.08	.35 (.37)	.89	1.42
	Socio-Economic Status (0 highest to 5 lowest)	-.34 (.31)	1.21	.70	-.30 (.31)	.90	.74
Reference Pattern «High» vs. Pattern «Middle»	Intercept	.88 (.75)	1.35	-	2.82** (.98)	8.32	-
	Gender (1 male; 2 female)	.12 (.26)	.21	1.06	-.22 (.34)	.43	.80
	Migration background (0 no MB, 1 with MB)	.11 (.27)	.18	.41	-.30 (.36)	.70	.73
	School Level (1 lower; 2 higher)	-.12 (.29)	.18	.81	-.39 (.38)	1.09	.67
	Socio-Economic Status (0 highest to 5 lowest)	-.22 (.25)	.79	1.12	-.45 (.32)	2.01	.63
Reference Pattern «Low» vs. Pattern «Middle”	Intercept	1.45 (.86)	2.82	-	2.82** (.98)	8.32	
	Gender (1 male; 2 female)	.06 (.31)	.03	1.06	-.22 (.34)	.43	.80
	Migration background (0 no MB, 1 with MB)	-.87* (.35)	6.24	.41	-.30 (.36)	.70	.73
	School Level (1 lower; 2 higher)	-.20 (.34)	.36	.81	-.39 (.38)	1.09	.67
	Socio-Economic Status (0 highest to 5 lowest)	.12 (.28)	.17	1.12	-.45 (.32)	2.01	.63
Pseudo-R-Quadrat	Cox und Snell	.037			.073		
	Nagelkerke	.043			.083		
	McFadden	.018			.037		

S.E. = Standard Error; OR = Odds Ratio;

*** *p* < .001;

** *p* < .01;

* *p* < .05,

^1^higher numbers indicate higher levels of the respective predictor

The socio-demographic variables showed for both waves (see Table 6) a very low prediction to the respective identified LCA patterns even if the explained variance for wave 1 was lower (wave 1 Cox and Snell = 3.7%) than for wave 2 (wave 2 Cox and Snell = 7.3%). At wave 1, having a migration background was associated with a significant and strong increase in the relative log odds of being in a low versus high well-being level pattern. This holds similarly for the relative log odds of being in a low versus middle well-being level pattern (see Table 6).

At wave 2, it was especially gender, not migration background as for wave 1 (see Table 6) that effected the highest prediction to the respective well-being patterns (see Table 6). At wave 2, being female was associated with a significant increase in the relative log odds of being in a low versus high and in a middle versus high well-being level pattern. Having a higher socio-economic status was associated at wave 2 with a higher probability of being in a higher versus middle well-being level pattern. At wave 2, only the effect of having a higher socio-economic status was associated with a higher probability of being in a middle versus low well-being level pattern.

## Discussion

Because latent class analysis (LCA) and LTA are exploratory and not confirmatory methods, we will address the findings with the needed containment. Using longitudinal questionnaire data from adolescents in Switzerland, analyses on adolescents’ school-related well-being and the respective changes in well-being during early adolescence were presented to understand possible changes over time. Following the most recent insights [13, 15, 26, 33, 48], we considered well-being a multidimensional construct that included both feeling good (hedonic concept) while performing academically positively and functioning well (eudemonic concept) at the individual level. Therefore, latent well-being patterns of adolescents at school were analyzed consisting of multiple indicators and their relations within the well-being classes as a latent construct.

Therefore, this study aimed to examine subjective adolescent students’ well-being patterns longitudinally by applying LCA and LTA. Using a multifaceted procedure, we expected to estimate the continuity of well-being levels and to explore whether transitions were to higher or lower well-being levels.

By applying six well-being indicators (life satisfaction, self-efficacy, self-esteem, self-determination, well-being, and satisfaction with grades at school), first a consecutive series of LCAs was applied to group students into empirically distinct well-being classes for the two study waves. For both waves, a three classes solution (low, middle, and high well-being pattern) was selected as to best fit the data. These analyses were crucial, as adolescence is a developmental stage with high risks in terms of psychological problems and adjustments related to subjective well-being [13] and significant decreases are assumed across the period of adolescence [47, 48]. The applied invariance analysis ensured both reliability for the identified number of well-being patterns (configural invariance), and established, as one of few studies so far, for both waves the same relevance of the three introduced well-being patterns (metric invariance) while ensuring structural and factorial comparability across both waves over a one-year period in early adolescence.

To model adolescent well-being transitions over time and following the methodological suggestions of Collins and Lanza [52], we identified significant differences in the longitudinal classification variables on the three well-being patterns (low, middle, and high level) via LTA. Considerable increases for the low well-being pattern and for the high well-being pattern were noticed, but a very high decrease of the middle pattern. Interestingly, and contrary to other studies often reported [47, 48, 49], we detected, based on these three well-being patterns, a very heterogeneous transition over time, although not a general decline on well-being over time for all adolescents. A striking stability (over 2/3) from wave 1 to wave 2 was identified for students showing a high well-being pattern. This contrasted the lower stability levels of the middle pattern with just about 40% over time. Regrettably, also the stability of the low well-being pattern was quite high over time with almost more than half of the students still being on the low well-being pattern. Thus, these results contribute to the debate on the decreasing-with-age issue: presumably, the assumption that all adolescents experience a decrease in well-being is not justified. It is possible that the inconsistent results and discrepancies in previous studies’ statements concerning the stability of the well-being in adolescence are due to not only methodological and contextual differences [18], but also the variance of developmental trajectories within a population of adolescents. It is conceivable that this decreasing trend only occurs in certain adolescent populations, e.g., only in the case of a confluence of unfavorable contextual factors, or that this trend begins at different set-points. As Casas et al. [48] have already shown with their analyses using different international samples, this trend may also have different set-points. Our participants were slightly older than 12 years old at the beginning of the study. Considering Casas et al.’s [48] results, indicating that the decreasing trend in most international samples occurred between the ages of 10 and 12, it cannot be excluded that this decreasing trend started before the age of 12 in some adolescent subgroups. Therefore, this may account for the discrepancy with other studies assuming a decreasing-with-age tendency.

Because well-being in adolescence is considered the backbone of mental health [33, 66–68], it is essential to understand how the dynamics of transitions over time have to be conceptualized and to which student groups the respective down- or upwards transitions apply. Therefore, for both waves we included socio-demographical covariates, such as gender, migration background, socio-economic status, and school level, that could plausibly relate to the well-being pattern variations at school and identified just a very low prediction to the respective identified patterns with the respective prediction for wave 1 being even lower than for wave 2. At wave 1, having a migration background was associated with a significant increase of being in a low versus high well-being level pattern. At wave 2, it was especially gender, not migration background, that showed the highest prediction to the respective well-being patterns. Contrary to Arpino and de Valk’s [69] or Safi’s [70] studies that found differences in reported subjective well-being between students with or without migration background, our results for early adolescents in Switzerland indicated no such important differences.

At wave 2, being female was associated with a highly significant increase of being in a low versus high and in a middle versus high well-being pattern. Gender, in the classical heteronormative dichotomy of being female or male, was not that distinctive as other studies suggested for well-being [7, 18, 71] or mental health in early adolescence [26, 28, 29, 56, 66]. Additionally, having a higher socio-economic status was associated at wave 2 with a higher probability of being in a higher well-being pattern.

The specific modeling of the calculations, as the way social reality is analyzed, has a distinctive role in the respective results in terms of mental health [10, 20, 66, 67]. Therefore, in further analyses in terms of identified well-being patterns, gender and other socio-demographical variables should be also considered [4]. Our latent models and identifying latent well-being patterns may be useful as an empirical basis for planning international explorations.

Well-being patterns in Swiss schools over time were identified. Well-being in adolescence also has to be addressed country specific and internationally [26, 59, 72] to develop international educational policies. Differences in adolescents’ lives are diverse and based on the respective culture [24, 49, 54, 55, 73].

Although we made a first step in assessing well-being data longitudinally, we are still cautious stating causal relations or even arguing about the overall phase of adolescence–as the two waves are only one year apart. As we measured at the beginning of each new school year, we do not know how the respective seasons might impact adolescent students’ reports of well-being. So far, there is no empirical evidence internationally of how these patterns could be different if assessed at additional timepoint in the middle of the respective school year. In middle and late adolescence, students’ well-being patterns could change over time, which does not just refer to the respective well-being levels [11], but also content wise to the indicators of which well-being consists of [3, 10].

Future research could run further analyses including aspects of resilience and mental health issues as indicators. Especially, when the question of fostering personal growth, school motivation and academic achievement, not just identifying, positive well-being development is the desideratum, promoting processes like supportive resilience factors and the role of specific mental health issues had to be further investigated and connected to the identified well-being classes. Furthermore, it would also be possible to test a similar model across three or more time points and test whether the predictors change over a longer period.

Lastly, it is also important to note that these findings should be considered within the context of COVID-19 because the data of wave 2 were collected in the later stages of the first COVID-19 wave. Researchers assessing survey data from the earlier stages of the first COVID-19 wave have pointed to an apparent increase in the frequency and severity of mental illness symptoms and a decline in psychological well-being for adolescents [74–76]. However, the results of some meta-analyses showed that the psychological impact of COVID-19 and its further consequences were small in magnitude [77–79]. More specific, the findings confirmed that the declines in psychological well-being and mental health were most pronounced during the pandemic’s early stages. Studies from the later stages of the first COVID-19 wave indicated a normalization in mental health conditions and an attainment of pre-pandemic levels [80]. Indeed, additional analyses conducted with the purpose of examining the changes in mental health outcomes using the same sample as the current study [78, 79] also revealed that the expected negative impact of the COVID-19 pandemic on mental health symptoms was not noticeable in the later stages of the first COVID-19 wave. Thus, we assume the initial increase in problems to be related to mental health and well-being reported at the onset of the pandemic may have not persisted in later stages of the first COVID-19 wave [81, 82]. Therefore, we believe our conclusions will hold true beyond the COVID-19 pandemic, even when being cautious because we still do not know how many COVID-19 waves are yet to come.

### Limitations

Additionally, because of sample restrictions, we could not statistically elaborate for the changes between the three levels because just three out of the six upwards and downwards paths consisted of sub-samples bigger than *n* = 25.

The attrition of about 18% between wave 1 and 2 was, considering the ongoing Covid-pandemic, low. Even though attrition for gender and migration-background was tested and no significant differences were found, we remain cautious on the two tested samples.

We did not account for the nested structure of the data (students embedded in 33 classes) as we chose a traditional LTA as a research method. Even more sophisticated research methods could be informative for reviewing the present findings. Additionally, because of the sample sizes (at wave 2 *n* = 319), the proportion of students in each class (six to 15 students), and the number of classes (33 classes) we did not run multilevel or similar analyses referring to class-specific results. For future research we suggest working with bigger sub-samples (about 450 students each wave) and from about 40 classes, to run multilevel analyses. Following Maas & Hox [83] and Akter & Khan [84], sampling for multilevel analysis is a challenging endeavour: Only bigger samples from at least 40 classes with at least 10 students in each class ensured robustness. Even if newer studies [85] comply with using smaller samples in multilevel analysis under certain conditions, the robustness is not considered as a given.

The results of our two-wave longitudinal analyzes will have to be verified as for full longitudinal analyzes at least three waves are needed [86].

Well-being is context-dependent and because of that, analyzes must be made context specific. Analyzes on adolescent students’ well-being were at the center of this study. This needed focus is also a limitation to the possibility of comparing well-being scores of different studies.

We used adolescents’ self-perceptions by the applied students’ questionnaires to understand their well-being at school. It would have been interesting to add teachers’ perceptions of the respective students because relations to teachers play a distinct role in adolescent students’ well-being [72]. The respective quality of the relationship between teachers and students, specifically teachers’ perceptions, expectations, and behaviors in the context of teaching and communication with their students are important predictors of students’ academic, personal, and social development [54].

Within this study, we could not test for a cut-off score of well-being. Due to this, it was not possible to test for the critical assumption that there might be an optimal, but not a maximal, level of well-being for the respective school classes.

## Conclusions

As adolescence is a phase of changes and transitions, it is crucial to understand how these changes and the connected risks can occur and how schools can support positive change. Especially when it comes to emotional and academical challenges in adolescence and the possibility of fostering positive development while minimizing risks as a valid resilience strategy [54], we need more knowledge for designing empirically validated prevention and intervention programs.

Our results indicate clearly that concerning the design of school-specific prevention and intervention programs, we must use a more complex approach focusing on specific students’ needs. Therefore, adolescents particularly on the low and middle well-being level need specific attention to support upward transitions to higher well-being level patterns and to mitigate downward transitions. As the old saying of ‘for to the one who has, more will be given’ holds for the high well-being level students’ group, the proverbial continuation of that same old saying, stating ‘…but from the one who has not, even what he has will be taken away’, applies distinctly for the middle and the low well-being levels in early adolescence.

## Supporting information

S1 Data (SAV)

## References

[1] Patalay P, Fitzsimons E. Development and predictors of mental ill-health and wellbeing from childhood to adolescence. Social psychiatry and psychiatric epidemiology. 2018;53(12):1311–1323. doi: 10.1007/s00127-018-1604-0 30259056

[2] Proctor C, Linley P, Maltby J. Youth Life Satisfaction: A Review of the Literature. Journal of Happiness Studies. 2009;10:583–630. doi: 10.1007/s10902-008-9110-9

[3] Hascher T., Hagenauer G. (2020). Swiss adolescents’ well-being in school. Swiss journal of educational research. 2020;42(2):367–390. doi: 10.24452/sjer.42.2.5

[4] Hascher T. Wellbeing. In: Peterson PL, Baker E, McGaw B., editors. International encyclopedia of education. 3rd ed. Oxford: Elsevier Ltd.; 2010. p. 732–738.

[5] Hascher T, Hadjar A. School alienation–Theoretical approaches and educational research. Educational Research. 2018;60(2):171–88. doi: 10.1080/00131881.2018.1443021

[6] Hatzichristou C, Lianos PG. (2016). Social and emotional learning in the Greek educational system: An Ithaca journey. International Journal of Emotional Education. 2016;8(2):105–127.

[7] González-Carrasco M, Casas F, Malo S, Viñas F, Dinisman T. Changes with age in subjective well-being through the adolescent years: Differences by gender. Journal of Happiness studies. 2017;18(1):63–88. doi: 10.1007/s10902-016-9717-1

[8] Compas BE, Jaser SS, Bettis AH, Watson KH, Gruhn MA, Dunbar JP, et al. Coping, emotion regulation, and psychopathology in childhood and adolescence: A meta-analysis and narrative review. Psychological bulletin. 2017;143(9):939. doi: 10.1037/bul0000110 28616996 PMC7310319

[9] Ryff CD. Psychological well-being revisited: Advances in the science and practice of eudaimonia. Psychotherapy and psychosomatics. 2014;83(1):10–28. doi: 10.1159/000353263 24281296 PMC4241300

[10] Dodge R, Daly AP, Huyton J, Sanders LD. (2012). The challenge of defining wellbeing. International journal of wellbeing. 2012;2(3). doi: 10.5502/ijw.v2i3.4

[11] Rees G, Goswami H, Bradshaw J. Developing an Index of Children’s Subjective Well-being in England. London: The Children’s Society, 2010. 16 p.

[12] Hajek A, König H. The role of optimism, self-esteem, and self-efficacy in moderating the relation between health comparisons and subjective well-being: Results of a nationally representative longitudinal study among older adults. British journal of health psychology. 2019;24(3):547–570. doi: 10.1111/bjhp.12367 30927338

[13] de la Barrera U, Schoeps K, Gil-Gómez JA, Montoya-Castilla I. Predicting Adolescent Adjustment and Well-Being: The Interplay Between Socio-Emotional and Personal Factors. International journal of environmental research and public health. 2019;16(23):4650. doi: 10.3390/ijerph16234650 31766641 PMC6926821

[14] Ryan RM, Deci EL. On happiness and human potentials: A review of research on hedonic and eudaimonic well-being. Annual review of psychology. 2001;52(1):141–166. Ryff CD. Psychological well-being revisited: Advances in the science and practice of eudaimonia. Psychotherapy and psychosomatics. 2014;83(1):10–28. doi: 10.1146/annurev.psych.52.1.141 11148302

[15] Ryff CD, Boylan JM, Kirsch JA. Disagreement about recommendations for measurement of well-being. Preventive Medicine. 2020;139:106049. doi: 10.1016/j.ypmed.2020.106049 32928444

[16] Hone L, Aaron J, Schofield G. An evaluation of positive psychology intervention effectiveness trials using the re-aim framework: A practice-friendly review. The Journal of Positive Psychology. 2015;10:303–22. doi: 10.1080/17439760.2014.965267

[17] Slee PT, Skrzypiec G. *Well-being*, *positive peer relations and bullying in school settings*. Switzerland: Springer, 2016.

[18] Motti-Stefanidi F, Pavlopoulos V, Mastrotheodoros S, Asendorpf JB. Longitudinal interplay between peer likeability and youth’s adaptation and psychological well-being: A study of immigrant and nonimmigrant adolescents in the school context. International Journal of Behavioral Development. 2020;44(5):393–403.

[19] Joshanloo M, Park YO, Park SH. Optimism as the moderator of the relationship between fragility of happiness beliefs and experienced happiness. Personality and Individual Differences. 2017;106:61–3. doi: 10.1016/j.paid.2016.10.039

[20] Turiano NA, Hill PL, Graham EK, Mroczek DK. Associations Between Personality and Health Behaviors Across the Life Span. In Ryff CD, Krueger RF, editors, The Oxford Handbook of Integrative Health Sciences. Oxford University Press. 2018. (Oxford Library of Psychology).

[21] Maccagnan A, Wren-Lewis S, Brown H, Taylor T. Wellbeing and Society: Towards Quantification of the Co-benefits of Wellbeing. Social Indicators Research. 2019 Jan 1;141(1):217–43. doi: 10.1007/s11205-017-1826-7

[22] Taylor RD, Oberle E, Durlak JA, Weissberg RP. Promoting positive youth development through school-based social and emotional learning interventions: A meta-analysis of follow-up effects. Child Development. 2017;88(4):1156–71. doi: 10.1111/cdev.12864 28685826

[23] Deci EL, Ryan RM. Facilitating optimal motivation and psychological well-being across life’s domains. Canadian Psychology/Psychologie Canadienne. 2008;49:14–23. doi: 10.1037/0708-5591.49.1.14

[24] Didaskalou E, Andreou E, Roussi-Vergou C, Skrzypiec G. Are Greek school students flourishing? null. 2018 Jul 3;36(3):223–237. doi: 10.1080/02643944.2018.1480185

[25] Childs AJ. Young Australians’ education and employment transitions: comparing young immigrants’ well-being to their Australian peers. null. 2018 Apr 3;10(2):121–138. doi: 10.1080/2005615X.2018.1460893 26. Cefai C, Simoes C, Caravita SCS. A systemic, whole-school approach to mental health and well-being in schools in the EU. 2021.

[26] Cefai C., Simoes C., & Caravita S. C. S. (2021). A systemic, whole-school approach to mental health and well-being in schools in the EU. Luxembourg: Publications Office of the European Union. doi: 10.2766/50546

[27] Duarte AC, Matos AP, Marques C. Cognitive emotion regulation strategies and depressive symptoms: gender’s moderating effect. Procedia-Social and Behavioral Sciences. 2015;165:275–283.

[28] Delaruelle K, Walsh SD, Dierckens M, Deforche B, Kern MR, Currie C, et al. Mental Health in Adolescents with a Migration Background in 29 European Countries: The Buffering Role of Social Capital. J Youth Adolesc. 2021;50(5):855–871. doi: 10.1007/s10964-021-01423-1 33791946

[29] Erol RY, Orth U. Self-esteem development from age 14 to 30 years: a longitudinal study. Journal of personality and social psychology. 2011;101(3):607.31. doi: 10.1037/a0024299 21728448

[30] Crocetti E, Moscatelli S, Kaniušonytė G, Meeus W, Žukauskienė R, Rubini M. Developing morality, competence, and sociability in adolescence: a longitudinal study of gender differences. Journal of Youth and Adolescence. 2019;48(5):1009–21. doi: 10.1007/s10964-019-00996-2 30778830

[31] Diener E, Emmons RA, Larsen RJ, Griffin S. The Satisfaction With Life Scale. Journal of Personality Assessment. 1985;49(1):71–5. doi: 10.1207/s15327752jpa4901_13 16367493

[32] Boon H, Kimhi S, Sapountzaki K, Parmak M, Ryan S, Groh A. Preliminary findings from an international study of subjective wellbeing in tertiary students. International Journal of Innovation, Creativity and Change. 2017;3:26–42.

[33] Bartels AL, Peterson SJ, Reina CS.Understanding well-being at work: Development and validation of the eudaimonic workplace well-being scale. PLoS ONE. 2019; 14(4): e0215957. doi: 10.1371/journal.pone.0215957 31022285 PMC6483236

[34] Goldbeck L, Schmitz TG, Besier T, Herschbach P, Henrich G. Life satisfaction decreases during adolescence. Quality of Life Research. 2007;16(6):969–979. doi: 10.1007/s11136-007-9205-5 17440827

[35] Joshanloo M. Investigating the relationships between subjective well-being and psychological well-being over two decades. Emotion. 2019;19(1):183–207. doi: 10.1037/emo0000414 29494203

[36] Suldo SM, Huebner ES. Is extremely high life satisfaction during adolescence advantageous? Social indicators research. 2006;78(2):179–203. doi: 10.1007/s11205-005-8208-2

[37] Ryff C, Singer B. Know Thyself and Become What You Are: A Eudaimonic Approach to Psychological Well-Being. Journal of Happiness Studies. 2008;9:13–39. doi: 10.1007/s10902-006-9019-0

[38] Deci EL, Ryan RM. A motivational approach to self: Integration in personality. In: Nebraska Symposium on Motivation, 1990: Perspectives on motivation. Lincoln, NE, US: University of Nebraska Press; 1991. p. 237–288. (Current theory and research in motivation, Vol. 38.). 2130258

[39] Deci EL, Ryan RM. The" what" and" why" of goal pursuits: Human needs and the self-determination of behavior. Psychological inquiry. 2000;11(4):227–268. doi: 10.1207/S15327965PLI1104_01

[40] Deci EL, Ryan RM. Autonomy and need satisfaction in close relationships: Relationships motivation theory. In: Weinstein N, editor. Human motivation and interpersonal relationships: Theory, Research, and Applications. Dordrecht: Springer Netherlands; 2014. p. 53–73.

[41] Bandura A. Self-efficacy: toward a unifying theory of behavioral change. Psychological review. 1977;84(2):191. doi: 10.1037//0033-295x.84.2.191 847061

[42] Bandura A. Self-efficacy: the exercise of control. New York: W.H. Freeman; 2012.

[43] Bandura A. Regulation of cognitive processes through perceived self-efficacy. Developmental psychology. 1989;25(5):729.

[44] Jerusalem M, Schwarzer R. Self-efficacy as a resource factor in stress appraisal processes. In: Schwarzer R, editor: Self-efficacy: Thought control of action. Washington, DC: Hemisphere; 1992. p. 195–213.

[45] Schwarzer R, Jerusalem M (1995). Generalized Self-Efficacy scale. In: Weinman J, Wright S, Johnston M, editors. Measures in health psychology: A user’s portfolio. Causal and control beliefs. Windsor, UK: NFER-NELSON; 1995. p. 35–37.

[46] Schwarzer R, Warner L. Perceived Self-Efficacy and its Relationship to Resilience. In: Resilience in Children, Adolescents, and Adults The Springer Series on Human Exceptionality. 2013. p. 139–50.

[47] Holte A, Barry MM, Bekkhus M, Borge AIH, Bowes L, Casas F, et al. Psychology of Child Well-Being. In: Ben-Arieh A, Casas F, Frønes I, Korbin JE, editors. Handbook of Child Well-Being: Theories, Methods and Policies in Global Perspective. Dordrecht: Springer Netherlands; 2014. p. 555–631.

[48] Casas F, González-Carrasco M. Subjective well-being decreasing with age: New research on children over 8. Child Development. 2019;90(2):375–394. doi: 10.1111/cdev.13133 30106474

[49] Tiliouine H, Rees G, Mokaddem S. Changes in self-reported well-being: A follow-up study of children aged 12–14 in Algeria. Child development. 2019;90(2):359–374. doi: 10.1111/cdev.13132 30125938

[50] Meuleners LB, Lee AH. Quality of Life Profile–Adolescent Version: Assessing the relationship of covariates to scale scores using structural equation modeling. Quality of Life Research. 2005;14(4):1057–1063. doi: 10.1007/s11136-004-2573-1 16041901

[51] Pancheva MG, Ryff CD, Lucchini M. An integrated look at well-being: Topological clustering of combinations and correlates of hedonia and eudaimonia. Journal of Happiness Studies. 2021;22(5):2275–97. doi: 10.1007/s10902-020-00325-6 34326680 PMC8315113

[52] Collins LM, Lanza ST. Latent class and latent transition analysis: With applications in the social, behavioral, and health sciences. Vol. 718. John Wiley & Sons; 2009.

[53] Hagenaars J, McCutcheon A. Applied latent class analysis. Cambridge University press. 2002.

[54] Kassis W, Govaris C, Chouvati R, Sidler P, Janousch C, Ertanir B. Identification and comparison of school well-being patterns of migrant and native lower secondary-school students in Greece and Switzerland: A multigroup latent profile analysis approach. International Journal of Educational Research. 2021;110:101863.

[55] Ullman C, Tatar M. Psychological Adjustment Among Israeli Adolescent Immigrants: A Report on Life Satisfaction, Self-Concept, and Self-Esteem. Journal of Youth and Adolescence. 2001;30(4):449–463. doi: 10.1023/A:1010445200081

[56] Gariepy G, Elgar FJ, Sentenac M, Barrington-Leigh C. Early-life family income and subjective well-being in adolescents. PLoS ONE. 2017;12(7): e0179380. doi: 10.1371/journal.pone.0179380 28715418 PMC5513414

[57] Kidger J, Araya R, Donovan J, Gunnell D. The effect of the school environment on the emotional health of adolescents: a systematic review. Pediatrics. 2012;129(5):925–49. doi: 10.1542/peds.2011-2248 22473374

[58] Moksnes UK, Moljord IE, Espnes GA, Byrne DG. The association between stress and emotional states in adolescents: The role of gender and self-esteem. Personality and individual differences. 2010;49(5):430–435. doi: 10.1016/j.paid.2010.04.012

[59] OECD. PISA 2009 Ergebnisse: Was Schülerinnen und Schüler wissen und können: Schülerleistungen in Lesekompetenz, Mathematik und Naturwissenschaften. Band 1. Bielefeld: Bertelsmann Verlag. Bielefeld, Rosenberg, M. (1965/2015). Society and the adolescent self-image. Princeton university press.

[60] Rosenberg M. Society and the adolescent self-image. Princeton university press; 2015.

[61] Makarova E. Akkulturation und kulturelle Identität: eine empirische Studie unter Jugendlichen mit und ohne Migrationshintergrund in der Schweiz. Vol. 8. Haupt Verlag AG; 2008.

[62] Weiss LA, Westerhof GJ, Bohlmeijer ET. Can we increase psychological well-being? The effects of interventions on psychological well-being: A meta-analysis of randomized controlled trials. PloS one. 2016;11(6):e0158092. doi: 10.1371/journal.pone.0158092 27328124 PMC4915721

[63] Lanza ST, Cooper BR. Latent class analysis for developmental research. Child Development Perspectives. 2016;10(1):59–64. doi: 10.1111/cdep.12163 31844424 PMC6914261

[64] Lanza ST, Bray BC, Collins LM. An introduction to latent class and latent transition analysis. In: Handbook of psychology: Research methods in psychology, Vol 2, 2nd ed. Hoboken, NJ, US: John Wiley & Sons, Inc.; 2013. p. 691–716.

[65] Muthén LK, Muthén BO. Mplus Version 8.6 User’s guide. Los Angeles, CA: Muthén & Muthén; 2020.66.

[66] who.int [Internet]. Adolsecent mental health; c2020 [cited 2021 Oct 29].

[67] Sawyer SM, Afifi RA, Bearinger LH, Blakemore S-J, Dick B, Ezeh AC, et al. Adolescence: a foundation for future health. The Lancet. 2012;379(9826):1630–1640. doi: 10.1016/S0140-6736(12)60072-5 22538178

[68] Reinhardt M, Horváth Z, Morgan A, Kökönyei G. Well-being profiles in adolescence: psychometric properties and latent profile analysis of the mental health continuum model–a methodological study. Health and Quality of Life Outcomes. 2020;18(1):95. doi: 10.1186/s12955-020-01332-0 32252785 PMC7137408

[69] Arpino B, de Valk H. Comparing life satisfaction of immigrants and natives across Europe: The role of social contacts. Social indicators research. 2018;137(3):1163–84. doi: 10.1007/s11205-017-1629-x 29962656 PMC5984636

[70] Safi M. Immigrants’ life satisfaction in Europe: Between assimilation and discrimination. European Sociological Review. 2010;26(2):159–76. doi: 10.1093/esr/jcp013

[71] Huppert FA, Whittington JE. Evidence for the independence of positive and negative well-being: Implications for quality of life assessment. British journal of health psychology. 2003;8(1):107–122. doi: 10.1348/135910703762879246 12643820

[72] Suldo SM, Friedrich AA, White T, Farmer J, Minch D, Michalowski J. Teacher Support and Adolescents’ Subjective Well-Being: A Mixed-Methods Investigation. 2009;38(1):67–85. doi: 10.1080/02796015.2009.12087850

[73] Park N. Life Satisfaction Among Korean Children and Youth: A Developmental Perspective. School Psychology International. 2005 May 1;26(2):209–223. doi: 10.1177/0143034305052914

[74] Duan L, Shao X, Wang Y, Huang Y, Miao J, Yang X, et al. An investigation of mental health status of children and adolescents in china during the outbreak of COVID-19. Journal of affective disorders. 2020;275:112–118. doi: 10.1016/j.jad.2020.06.029 32658812 PMC7329661

[75] Jones EA, Mitra AK, Bhuiyan AR. Impact of COVID-19 on mental health in adolescents: a systematic review. International journal of environmental research and public health. 2021;18(5):2470. doi: 10.3390/ijerph18052470 33802278 PMC7967607

[76] Magson NR, Freeman JY, Rapee RM, Richardson CE, Oar EL, Fardouly J. Risk and protective factors for prospective changes in adolescent mental health during the COVID-19 pandemic. Journal of youth and adolescence. 2021;50(1):44–57. doi: 10.1007/s10964-020-01332-9 33108542 PMC7590912

[77] Nearchou F, Flinn C, Niland R, Subramaniam SS, Hennessy E. Exploring the impact of COVID-19 on mental health outcomes in children and adolescents: a systematic review. International journal of environmental research and public health. 2020;17(22):8479. doi: 10.3390/ijerph17228479 33207689 PMC7698263

[78] Prati G, Mancini AD. The psychological impact of COVID-19 pandemic lockdowns: a review and meta-analysis of longitudinal studies and natural experiments. Psychological Medicine. 2021;51(2):201–211. doi: 10.1017/S0033291721000015 33436130 PMC7844215

[79] Robinson E, Sutin AR, Daly M, Jones A. A systematic review and meta-analysis of longitudinal cohort studies comparing mental health before versus during the COVID-19 pandemic. medRxiv. 2021. doi: 10.1101/2021.03.04.21252921 PMC8578001 34600966

[80] Rogers AA, Ha T, Ockey S. Adolescents’ perceived socio-emotional impact of COVID-19 and implications for mental health: results from a US-based mixed-methods study. Journal of Adolescent Health. 2021;68(1):43–52. doi: 10.1016/j.jadohealth.2020.09.039 33143986 PMC7605752

[81] Xie X, Xue Q, Zhou Y, Zhu K, Liu Q, Zhang J, et al. Mental health status among children in home confinement during the coronavirus disease 2019 outbreak in Hubei Province, China. JAMA pediatrics. 2020;174(9):898–900. doi: 10.1001/jamapediatrics.2020.1619 32329784 PMC7182958

[82] Zhou S-J, Zhang L-G, Wang L-L, Guo Z-C, Wang J-Q, Chen J-C, et al. Prevalence and socio-demographic correlates of psychological health problems in Chinese adolescents during the outbreak of COVID-19. European Child & Adolescent Psychiatry. 2020;29(6):749–758. doi: 10.1007/s00787-020-01541-4 32363492 PMC7196181

[83] Maas C. J., & Hox J. J. Robustness issues in multilevel regression analysis. Statistica Neerlandica. 2004; 58(2): 127–137.

[84] Akter N. J., & Khan M. H. R. Effect of Sample Size on the Profile Likelihood Estimates for Two-stage Hierarchical Linear Models. Journal of Biomedical Analytics. 2018; 1(2): 81–89.

[85] Hox J., & McNeish D. Small samples in multilevel modeling. Small sample size solutions, 2020. Routledge: 215–225.

[86] Ployhart R. E., & MacKenzie W. I. (2015). “Two waves of measurement do not a longitudinal study make,” in More statistical and methodological myths and urban legends, eds Lance C. E. and Vandenberg R. (New York, NY: Routledge), 85–99.

